# Surgery

**Published:** 1894-12

**Authors:** James Swain


### SURGERY.

The surgery of the ureters, which is one of the most recent triumphs of surgical art, forms the subject of an able article[1] by Dr. Christian Fenger, in which the various operations which have been performed on these ducts are briefly considered. The ureter can be reached by the trans-peritoneal and the extra-peritoneal routes; but other things being equal, the latter method, in spite of the greater difficulty arising from the depth of the wound, should be adopted, as the risk of peritonitis is thereby avoided. The intra-peritoneal operation is best carried out by means of a median or lateral abdominal incision, by which the entire course of the ureter can be reached: the extra-peritoneal method differs according to the part of the ureter requiring operation; the upper two-thirds being accessible by a continuation of the ordinary oblique incision for lumbar nephrotomy from the twelfth rib downwards along the ilium and Poupart's ligament to about the middle of the latter, the lower third or pelvic portion being reached by a lateral sacral incision as proposed by Delbet,[2] or better by Kraske's[3] operation, or the osteo-plastic resection of the sacrum as proposed by Cabot.[4]

Accidental wounds and subcutaneous ruptures of the ureter have not yet been dealt with surgically. The reason for this is that diagnosis cannot be effected sufficiently early, owing to the uncertainty of the symptoms; for hæmaturia may be slight or absent, and swelling from the accumulation of extravasated urine is not seen for one or several weeks after the injury. It will be advisable, however, as soon as the diagnosis can be made, or when lumbar opening of a peri-ureteral cavity containing urine is made, to look for the seat of rupture and endeavour to restore the continuity of the tube.

Stones in the ureter are generally arrested in the upper portion, and with about equal frequency in the middle and vesical portions. According to their position they can be removed:—(*a*) Through the bladder by dilatation of the female urethra, or by suprapubic cystotomy; in short, so far as operation is concerned, they may be classed among stones in the bladder. (*b*) Ureterotomy through the rectum.[5] (*c*) Ureterotomy through the vagina.[6] (*d*) Extra-peritoneal ureterotomy.[7] (*e*) Intra-peritoneal ureterotomy.[8] The locality of the stone has only been diagnosed before operation in the cases in which the stone was afterwards removed through the vagina or rectum. Extra-peritoneal ureterotomy for stone has been done in five cases, all of which were successful; and of two cases treated by the

1 *Ann. Surg.*, vol. xx., 1894, p. 257.
2 *Ann. d. mal. d. org. génito-urin.*, 1891, p. 246.
3 *Ann. Surg.*, vol. ii., 1885, p. 415. 4 *Am. J. M. Sc.*, vol ciii., 1892, p. 43.
5 *Riforma med.*, Sept. 5, 1887. 6 *Boston M. & S. J.*, Dec. 25, 1890.
7 *Chicago M. Recorder*, March, 1893. 8 *Lancet*, 1890, vol. ii.

intra-peritoneal method, one was successful and one died from peritonitis. Fenger therefore argues that uretero-lithotomy by the extra-peritoneal route is a safe operation. The wound heals without stenosis; and suturing is unnecessary, for deep drainage of the wound is sufficient. Intra-peritoneal ureterotomy should be done only when access outside of the peritoneal cavity is impossible, and should be completed by careful suturing, covering with a peritoneal or omental flap, and drainage. Opening the peritoneal cavity for diagnostic purposes may occasionally be necessary, and when this has been done the abdomen should be closed and the stone removed through an extra-peritoneal incision. Operation for valve-formation (which always causes an intermittent or permanent impediment to the flow of urine, leading to hydro- or pyo-nephrosis) is best effected by the extra-peritoneal lumbar incision. The dilated pelvis or hydronephrotic sac is easily found and opened by a longitudinal incision. If the opening of the ureter into the sac cannot be found, it may be located by incising the ureter below the sac and passing a probe upwards towards the pelvis. The valve or inner wall of the ureter, running in the sac, is now divided longitudinally from the opening in the sac, and the resultant wound treated in one of the following ways: (*a*) By turning the flaps out and uniting them to the inner wall of the sac by sutures (Trendelenburg).[1] (*b*) By drawing the corners of the longitudinal incision together with one suture, transforming the longitudinal into a tranverse wound (Fenger).[2] (*c*) Uniting the wound longitudinally with numerous fine silk sutures, avoiding the mucous membrane (Mynter).[3] The cases recorded by Fenger and Mynter were successful. Compression of the ureter for diagnosis of kidney affections has not been attended with any degree of success, and has been replaced by catheterisation.

Catheterisation of the ureters from the bladder has reached a state of practical usefulness only in women, and has occasionally given valuable information affecting the decision for or against operation on the kidney. In man, however, catheterisation is practicable only through epicystotomy; but as the mortality of this operation is steadily decreasing, the procedure may be said to be justifiable in selected cases. Ureteral catheterisation from the bladder for the evacuation of hydro- or pyo-nephrosis has occasionally been performed successfully (Pawlik). It is probable that only strictures in the upper portion of the ureter are accessible for operative interference, although Kelly[4] met with temporary success in one case of stricture low down by passing elastic bougies from the bladder. Such strictures have been treated in three ways: (*a*) Dilatation by bougies from the pelvis of the kidney as reported by Alsberg.[5] (*b*) By longitudinal

[1] *Samm. klin. Vortr.*, No. 355, 1890.
[2] *J. Am. M. Ass.*, vol. xxii., 1894, p. 335. [3] *Ann. Surg.*, vol. xviii., 1893, p. 658.
[4] *Johns Hopkins Hosp. Rep.*, vol ii., 1891, p. 235.
[5] *Verhandl. d. deutsch. Gesellsch. f. Chir.*, xxi. Congres, 1892, p. 43.

division of the stricture and transverse union of the wound as practised by Fenger.[1] This operation is on the plan of the Heinicke-Mikulicz operation for stenosis of the pylorus, and is useful for strictures which are not too extensive. The upper and lower ends of the ureter are brought together by folding the ureter upon itself, and the remainder of the wound is united by sutures through the outer and middle coats. (*c*) Resection of the ureter, and implantation of the distal end into the pelvis of the kidney as practised by Kuster,[2] who, after transverse division of the ureter below the stricture and at the entrance to the pelvis of the kidney, unites the ureter to the pelvis by dividing the upper end of the ureter in a longitudinal direction, unfolding the divided end, and suturing it to the opening in the sac—the remainder of the wound being closed by catgut sutures. Fenger thinks this operation may be useful in rupture or division or stricture of the upper end of the ureter; but in a similar case of stricture in the upper end of the ureter, especially if the ureter were not elongated or the kidney movable, he would prefer the plastic operation he has proposed *(vide supra)*, as it is easier of technique and has proved successful in a case of traumatic stricture below the pelvic orifice. Longitudinal wounds of the ureter have already been considered in the discussion of the operation for stone. Transverse wounds are much more difficult to deal with satisfactorily, as there is a tendency to retraction of the wound, causing the sutures to tear out, and there is always a liability to stenosis; in experiments upon animals and in operations on the human subjects attempts to unite complete tranverse wounds of the ureter have failed. Complete transverse wounds of the upper end of the ureter should be treated by implantation of the ureter into the pelvis of the kidney (Kuster's operation, *vide supra*); those near the bladder should be treated by implantation into that viscus; and those in the continuity of the ureter should be treated by uretero-ureterostomy, after Van Hook's[3] method of lateral implantation if possible. Van Hook's method of anastomosis is as follows: (*a*) Ligate the lower portion of the tube one-eighth or one-fourth of an inch from the free end, with silk or catgut. Make with fine, sharp-pointed scissors a longitudinal incision, twice as long as the diameter of the ureter, in the wall of the lower end, one-fourth of an inch below the ligature. (*b*) Make an incision, with the scissors, in the upper portion of the ureter, beginning at the open end of the duct, and carrying it up one-fourth of an inch. This incision insures the patency of the tube. (*c*) Pass two small cambric sewing needles, armed with one thread of sterilised catgut, through the wall of the upper end of the ureter, one-eighth of an inch from the extremity, from within outwards, the needles being from one-sixteenth to one-eighth of an inch apart, and

[1] *J. Am. M. Ass.*, vol. xxii., 1894, p. 335.
[2] *Arch. f. klin. Chir.*, band xliv., heft 4, 1892.
[3] *J. Am. M. Ass.*, vol. xxi., 1893, pp. 911, 965.

equidistant from the end of the duct. It will be seen that the loop of catgut between the needles firmly grasps the upper end of the ureter. (*d*) These needles are now carried through the slit in the side of the lower end of the ureter into and down the tube for half-an-inch, where they are pushed through the walls of the duct, side by side. (*e*) It will now be seen that the traction upon this catgut loop passing through the wall of the ureter will draw the upper end of the duct into the lower portion. This being done, the ends of the loop are tied together securely, and, as the catgut will be absorbed in a few days, calculi do not form to obstruct the passage of the urine. (*f*) The ureter is now enveloped carefully with peritoneum, as already described in other operations, provided an intra-peritoneal operation has been done.

In addition to this procedure, Bloodgood[1] and others have applied two sutures through the external coats only, as an additional security against leakage. This operation has been successfully performed on the human subject by Kelly,[2] who accidentally divided the ureter whilst removing a fibroid of the uterus.

Division of ureter, with loss of substance so considerable as to make reunion of the two ends impossible, requires different operative procedures:—(*a*) Implantation of the ureter into the bladder. This operation is preferable to all other procedures when the upper end of the divided ureter is long enough to reach the bladder; and may be performed either by extra- or intra-peritoneal methods. Paoli and Busachi[3] suggest that this operation should be done by splitting the distal end of the ureter, and uniting it by sutures to an incision in the bladder; but Penrose[4] and others have acted upon Van Hook's suggestion of uretero-ureterostomy, and in a similar manner invaginated the cut end of the ureter into the bladder. (*b*) Implantation of the ureter into the bowel. This operation is strongly condemned by Van Hook on account of the liability to infection of the ureter and kidney. The bowel therefore should never be chosen when it is possible to implant the ureter into the bladder. If this be impossible, on account of defect in the ureter, it is still an open question whether or not implantation into the colon should be tried before resorting to implantation in the lumbar region, or abdominal wall, or to nephrectomy. (*c*) Implantation of the ureter on the skin. Losses of substance of the ureter, too extensive to permit of uretero-ureterostomy, or located too high up to permit of implantation of the upper end into the bladder, will require either implantation on the skin or into the bowel; but the latter is objectionable for the reason already given. Implantation on the skin may have to be followed by secondary nephrectomy,

[1] *Ann. Surg.*, vol. xix., 1894, p. 77.
[2] *Ibid*, p. 70. [3] *Ann. d. mal. d. org. génito-urin.*, 1888.
[4] *Med. News*, vol. lxiv., 1894, p. 470.

which, however, appears to be much less dangerous than the primary operation.

The prevention of incontinence of urine caused by uretero-uterine and uretero-vaginal fistulæ is accomplished in three ways:—(*a*) Plastic operations, with a view to displace the fistula from the vagina or cervix into the bladder. In most of the cases, however, it will be better to perform an implantation of the ureter into the bladder. (*b*) Kolpokleisis, which consists in closure of the vagina on the distal side of a vesico-vaginal fistula. The disadvantages of this operation are that the fistula may contract, and that marital relations are made impossible except in cases where a partial kolpokleisis can be made. (*c*) Nephrectomy, the operation of last resort. It has been necessitated in some instances by infection of the kidney; and although in fourteen cases only one patient died, it is applicable only to cases where the other kidney is healthy.

Dr. Fenger gives an account of forty cases of uretero-vaginal fistula, of which two were secondary to operation on vesico-vaginal fistula, three followed pelvic abscesses, five occurred after vaginal hysterectomy, twenty-eight followed childbirth, and in two the cause was unknown. Of these forty, twenty-four were operated upon as follows: Plastic invagination into the bladder through the vagina was successful in ten cases; kolpo-kleisis and nephrectomy were each performed in five cases; and in the remaining four, attempts at operation were abandoned as unsuccessful.

JAMES SWAIN.

